# Drugging the undruggable: activity-based protein profiling offers opportunities for targeting the KLK activome

**DOI:** 10.1080/15384047.2022.2033059

**Published:** 2022-02-06

**Authors:** Kristi Y. Lee, Cindy H. Chau, Douglas K. Price, William D. Figg

**Affiliations:** Molecular Pharmacology Section, Genitourinary Malignancies Branch, Center for Cancer Research, National Cancer Institute, National Institutes of Health, Bethesda, MD, USA

**Keywords:** Prostate cancer, kallikrein, KLK activome, activity-based protein profiling, proteomics

## Abstract

The vast majority of the human proteome is yet to be functionally characterized thus hindering ongoing investigations on potential drug resistance mechanisms and advanced treatment options. Chemical proteomics is a powerful solution for enzyme profiling and the development of next generation cancer therapeutics previously deemed undruggable by small molecules. Within this field, activity-based protein profiling (ABPP) is a specialized technology capable of discriminating enzyme interactions that occur within complex, biological environments. In a recent publication by Lovell et al, the kallikrein-related peptidase (KLK) family of serine proteases that is highly implicated in the progression of prostate cancer (PCa) was subject to ABPP to elucidate enzymatic activities in the presence of enzalutamide. This is the first report of ABPP in PCa and of activity-based chemical probes selective for individual KLKs. Further, the study reveals androgen receptor-dependent activity among KLK proteins, particularly in mediating the invasion of the bone microenvironment.

What was once considered the undruggable proteome is a world of therapeutic potential due to functional assays powered by chemical proteomics. Among these assays, activity-based protein profiling (ABPP) is a cutting-edge technology designed to broadly classify direct enzyme activities that are dependent on the dynamic biological environments in which they exist.^1^ The utility of ABPP high-throughput drug screens lies in the activity-based chemical probes which act as precursors for the design and optimization of bioactive small molecule inhibitors. ABPP can use small-molecule chemical probes to elucidate the mechanisms behind drug-target interactions or identify active sites of target proteins. These preclinical proteomics studies already have applications in the study of melphalan resistance in multiple myeloma, target discovery for dasatinib in gastric cancer, and curcumin resistance screening in colon cancer among many functional ABPP-driven investigations.^2–4^ From a clinical perspective, these assays may be better equipped at predicting off target effects well before entering clinical trials as protein interactions are often synergistic across multiple biological pathways.^5^ Chemical proteomics has aided in the characterization of enzyme activity profiles across multiple enzyme families such as proteases, phosphatases, glycosidases, oxidoreductases, and kinases further demonstrating the abundance of targeting potential from a drug development standpoint.^6^ Given the dynamic nature of protein interactions driving multiple disease pathways at once, functional proteomics is a vital component of drug discovery beyond the genomic and post-transcriptional level.

A recent article by Lovell et al. presented novel findings on the largely uncharacterized activity profiles of the Kallikrein-related peptidase (KLK) family of serine proteases, a network referred to as the KLK activome and implicated in prostate cancer (PCa) growth, progression and metastasis.^7^ Aberrant expression of KLKs and their dysregulation are implicated in many solid tumors, including breast, ovarian, and pancreatic cancers, thus making KLKs attractive biomarkers of disease and emerging therapeutic targets.^8^ However, to date the development of this potential target has been hampered by the lack of technology for specific assessment of KLK activity within the complex KLK activome. Lovell et al. is the first reported design and application of activity-based probes (ABPs) capable of discriminating activities between individual KLK proteins. Further, the study is the first in PCa to investigate mechanisms of drug resistance using ABPP. Gel-based activity profiling determined that KLK2 and KLK3 are highly activated in the presence of dihydrotestosterone (DHT) and KLK14 is deactivated by DHT but rescued by the advanced PCa drug and androgen receptor (AR) antagonist, enzalutamide. The KLK14 response to DHT and enzalutamide likely proposes a functional component as to the resistance mechanisms of AR inhibitors; additional assays also show that active KLK14 is secreted from both osteoblasts and PCa cells to promote tumor cell migration. Co-culturing of osteoblasts and LNCaP cells revealed cross-reactivity of KLK14 with KLK2 and KLK3 which may further intensify osteoblast formation and metastasis. Prominently, the study found that KLK activity is dysregulated from expression as total KLK protein quantities were more abundant than quantities from ABPs. This suggests that measuring KLK proteolysis can be coupled together with routine prostate specific antigen (PSA) testing for improved PCa diagnosis and monitoring, highlighting a role for the use of ABPs to assess KLK activity as a novel PCa biomarker.

ABPP is dependent on optimized probes which typically are derived from known inhibitors or natural products specific to catalytic class.^9^ The active site-specific component of the probe consists of an electrophilic, reactive “warhead” that irreversibly binds a protein active site.^10^ These reactive sites are linked to molecules mimicking the active site’s natural substrate to further facilitate the covalent interaction through active site stabilization. In contrast to other inhibitors, ABPP chemical probes are typically bound to reporter tags to quantify binding interactions through either gel-based fluorescence or biotin-labeling for affinity-tagged blotting.^9^ Other more advanced techniques exist as well, such as in the case of isoTOP-ABPP, which utilizes isotopically labeled probes for quantification via mass spectrometry.^11^ Thus, using this three-part formula, the authors successfully optimized orthogonal chemical probes for the simultaneous characterization of KLK2, KLK3, and KLK14. As characterizing individual protein activities within disease pathways is complex, Lovell et al.’s findings are noteworthy, as they demonstrate that inhibiting specific KLK proteases further activates proteolysis and disease progression. The KLK enzyme family previously had no known reports of selectivity for small molecule inhibition, thus limiting its potential as a therapeutic target and biomarker candidate. The novel findings in Lovell’s study on selective KLK probes may inspire the future of ABPP-driven small molecule drug discovery not only in PCa but other malignancies as well. For instance, KLK4 has been previously published in ovarian cancer and ABPP probe development may pertain to inhibitor and small molecule optimization for this disease.^12,13^

Acquired drug resistance remains a challenge for patients in the setting of castration-resistant disease who are refractory to next-generation targeted therapies. Dissecting the KLK activome has improved the understanding of its role in PCa and reveal new opportunities on how to overcome mechanisms of bone metastasis and drug resistance. Characterization of individual KLK activity, clinical utility of ABPP and related proteomic studies have strong potential to drive PCa biomarker discovery of active enzymes to supplement traditional prognostic strategies such as PSA marker screening as well as PROTAC design from a therapeutic standpoint. In fact, ABPP screens have already aided in the discovery of small molecule recruiters of E3 ligases to optimize PROTACs for the improved degradation of the bromodomain heteroprotein, BRD4.^14^ The possibilities of expanding the previously deemed undruggable proteome are broad given these technologies and future work will greatly aid in the optimization of therapies that are dependent on complex biological contexts.
